# Direct anterior approach in total hip arthroplasty: more indications and advantages than we found

**DOI:** 10.1186/s42836-022-00130-x

**Published:** 2022-07-08

**Authors:** Zhonghua Xu, Jun Zhang, Jie Li, Yuan Zhang

**Affiliations:** Joint Disease & Sport Medicine Center, Department of Orthopedic Surgery, Xinqiao Hospital, Army Medical University, 183 Xinqiao street, Shapingba District, Chongqing, 400038 China

**Keywords:** Direct anterior approach, Total hip arthroplasty, Indications, Advantages, Hip-spine relation

## Abstract

Mounting attention has been oriented to the direct anterior approach (DAA) in total hip arthroplasty (THA) because of alleged advantages in terms of tissue-sparing and minimal invasiveness, as well as fast recovery after surgery in the past decades. Doubt has also been raised by critics regarding learning curve, indications, technical feasibility, intraoperative risks and complications, and unconfirmed long-term consequences of the approach. The controversies were elaborately reviewed and discussed in a recent publication in *Arthroplasty* by Realyvasquez *et al*. from the perspective of American surgeons. This inclusive review covered the current status, technical issues, outcome evaluation, and developmental concerns of DAA in modern THA. As one of the pioneers of DAA in hip surgery communities in China, Prof. Y. Z., the corresponding author of the paper, has his own understanding and manipulation of DAA on the basis of thousands of DAA procedures he has performed. The purpose of this article was to respond to the pivotal issues discussed in the article by Realyvasquez *et al*., and to present our own view of points about the indications/contraindications and advantages as different from existing ideas. In particular, we proposed a Xinqiao Predictive Algorithm to quantitatively assess the indications for and feasibility of DAA for the first time. The algorithm was constructed on multiple factors derived from Chinese patients. Our study concluded that the potential advantages of DAA could be achieved by personalizing the pelvic functional position and placing the component into the real safe zone, by means of adapting to the spaciotemporal change of the hip-spine coordination.

## Background

A recent publication in *Arthroplasty* titled “The direct anterior approach to the hip: a useful tool in experienced hands or just another approach?” by Realyvasquez *et al*. comprehensively discussed the current status, surgical technical issues, outcome evaluation, and developmental concerns of direct anterior approach (DAA) in total hip arthroplasty (THA) [1]. It provided added value for both the beginners and specialists of hip arthroplasty. However, we are still not sure what the answer is to the question raised in the title of this article after careful reading: Is it a tool for experienced hands or just another approach?

All controversies on surgical approaches in THA are, in essence, about the clinical efficacy and long-term outcomes. The former has been well substantiated by multiple studies. A meta-analysis, published on *Bone & Joint Journal* in 2021, reviewed 29 clinical trials (7 Level I RCTs, 7 Level II prospective observational studies, and 15 Level III retrospective observational studies), which compared DAA and posterolateral primary THA, and found that the use of DAA, not the posterolateral approach (PA), had earlier benefits in terms of functional recovery and pain relief. However, these benefits were short-lasting, with no significant differences seen in later follow-up studies (6 weeks postoperatively, in light of the Harris Hip Score and the 12-Item Short Form Survey physical score, and 8 weeks after operation, as rated on the University of California and Los Angeles activity scale) [2]. The deficiencies included increased cumulative cost for hospital treatment and blood transfusion, and minimal but significant risk of major surgical consequences, including infection, dislocation, readmission, emergency treatment, and revision surgery according to a retrospective cohort study published on *JAMA Surgery* in 2020, which included 5986 propensity-score matched patients and compared DAA with posterior or lateral approach [3].

Although, to date, no evidence has proved the improved long-term outcomes, increasing data have revealed the good-to-excellent mid-to-long-term clinical outcomes of DAA compared to other approaches. A retrospective study conducted in South Africa assessed the result of DAA over an average time of 7.35 years using patient-reported outcomes. Thirteen revisions were performed among all the 522 patients, and the overall 5-year implant survival rate was 97.5%. Patient joint perception scores showed that 65.5% of the subjects perceived a completely natural joint. Median Forgotten Joint Score-12 was 90 and modified Harris Hip Score was 88 [4]. Another retrospective study in Switzerland that followed up 256 patients for a minimum of 10 years reported 9 revision procedures, and the overall implant survival rate was 96.8% 10 years after the surgery. The median subjective hip value was 90%, and the Western Ontario and McMaster Universities Osteoarthritis Index score reached a median of 0.2 points [5].

As one of the pioneers of DAA in hip communities in China, the corresponding author (Y. Z.) and his colleagues in Xinqiao Hospital have performed over 3000 cases of primary THA via DAA since 2013, including more than 500 cases of complex primary THA, which involved complicated pathologies including high-dislocated hip dysplasia (Crowe type III-IV), hip infection sequela, stiff or ankylosed hips caused by various pathologies. We also developed several innovative techniques in terms of targeted soft-tissue releasing, leg-length equalization algorithm, and subtrochanteric shortening osteotomy (SSO) [6–8]. The results showed superior functional recovery in general and follow-up studies exhibited improvement in gait and hip abduction and flexion muscle strength in particular [9, 10]. Furthermore, in the recent two years, we also explored the clinical potential of extensile DAA in 50 cases of partial and total revisions in hip arthroplasty. Presented here are our understanding and opinions regarding some major topics covered by the article by Realyvasquez *et al*.

### Current status and outlook of DAA in China

With mounting interest in tissue-sparing and small-incision arthroplasty, the use of DAA has been on the steep rise across the globe. Chinese clinicians also jump to the bandwagon of DAA, which is now popular in the U.S. While DAA application in the U.S. and some Europe countries has been well documented [11, 12], official data on DAA are still scanty in China, partially due to the late introduction of the Chinese joint registry program. An informal investigation among the members of Chinese Hip Society and Chinese Orthopedic Elite Club revealed that the total volume of DAA in China registered a triple-fold growth in 2021 (nearly 40,000), as compared to 2015. However, the percentage of DAA, against the total volume of THA, is less than 10%, which is significantly lower than that of the U.S (approximately 40%). Moreover, half of DAA were done in the tier-II county hospitals, according to a survey conducted among hip surgeons practicing in Sichuan Province and Chongqing City, indicating a relatively slow progress in hip surgery in China. However, our estimation is that the DAA rate against total THA will be on a par with the average level of Europe and the U.S. in not-very-distant future, with the authorization of the charge in terms of special value and medical service of DAA from the government.

### Transformable indications and contraindications for DAA

It seems that general consensus has been reached regarding the indications and contraindications of DAA by previous studies [13, 14]. Admittedly, surgeons at beginner level should be fully aware of the indications and contraindications for DDA to prevent iatrogenic complications. Nonetheless, too much emphasis on this issue would inevitably hinder the widespread application of this innovative surgical idea and approach. In Realyvasquez’s article, the authors believe that high BMI (>30 kg/m^2^), specific pelvic anatomic variability and more comorbidities (>3) are contraindications to DAA. This conclusion is premised on the finding that high BMI is indeed a risk factor affecting the smooth implementation of DAA. However, the decision of whether a high BMI is contraindicated to DAA or not, should be based on the cause of the high BMI and stratification of risk factors.

To be specific, if a high BMI is caused by increased muscle mass in male and training population, a routine surgical procedure, with or without targeted soft tissue releasing, will be sufficient to avoid high-BMI-related complications. On the other hand, if a high BMI is the result of excessive fat accumulation, complication and reoperation rates were higher in the high-BMI population than in their counterparts with a normal BMI [15]. In this scenario, concurrent pathologies in combination with other risk factors that affect wound healing, such as diabetes mellitus, hypothyroidism, dermatitis, and other microvascular insufficiency disorders, will render the high BMI a contraindication to DAA. Furthermore, cardiopulmonary comorbidities also constitute a contraindication to supine DAA in patients with large abdominal panniculus, since the compression of massive panniculus onto chest dramatically increases the airway resistance, cardiovascular load and intraoperative risks.

On the basis of aforementioned analysis, we are led to a conclusion that a high BMI alone is not a definitive contraindication for DAA. Since waist-hip ratio (WHR) has been proved to be a better indicator of abdominal obesity than BMI by World Health Organization [16], we also advocate use of this index, which, we believe, can better indicate the impact of fat mass on surgical manipulation and wound healing.

**Fig. 1:**
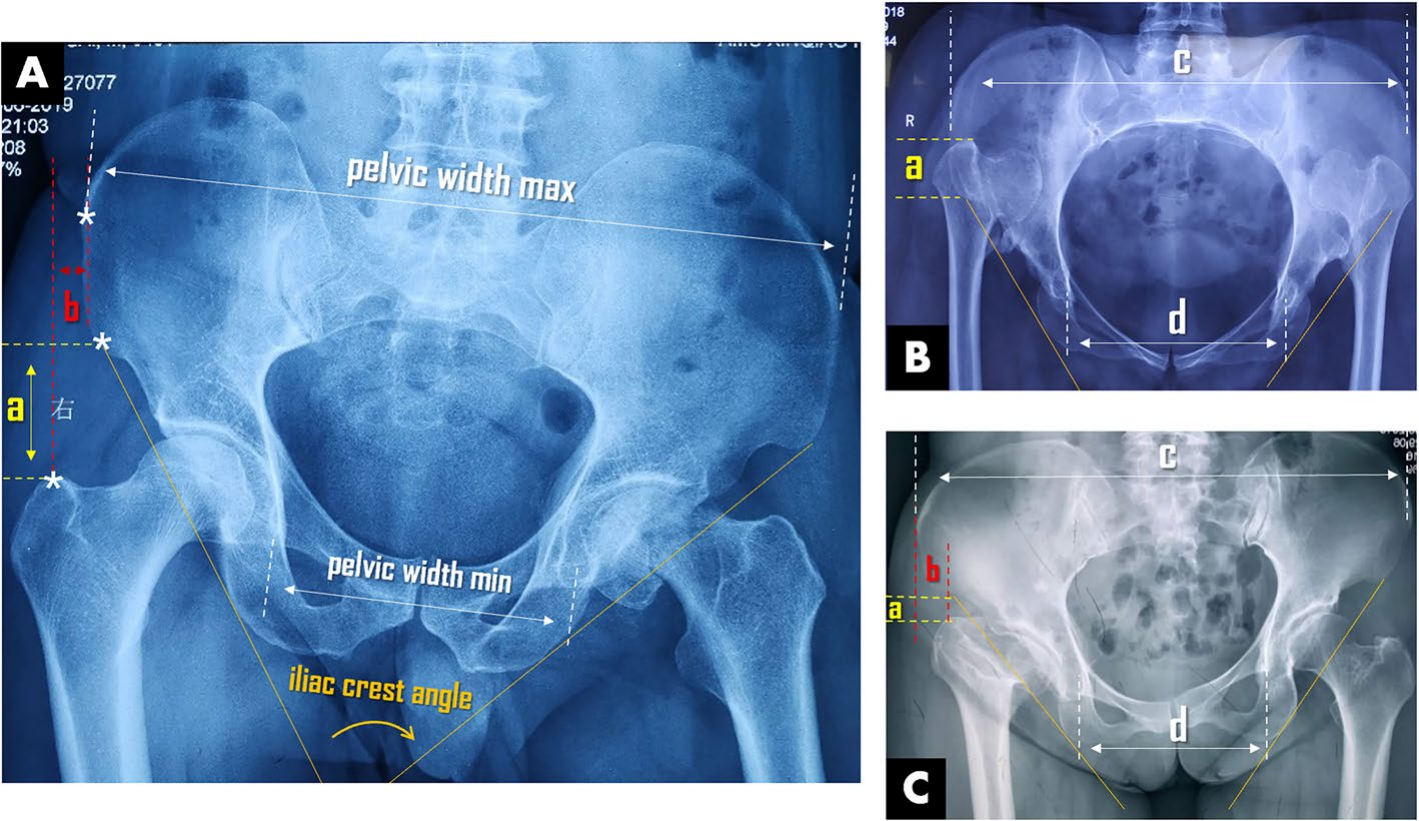
Pelvic anatomy-related indicators for evaluating feasibility of DAA and their measurement on a standard anteroposterior pelvic X-ray. **A** Left femoral neck fracture in a pelvis with normal anatomy related-index is strongly indicated for both DAA beginner and expert. **B** Bilateral high-dislocated developmental dysplasia of the hip in a pelvis with abnormal anatomy related-index is conditionally indicated for DAA expert. **C** Infection sequela of the right hip in a pelvis with abnormal anatomy related-index is fairly indicated for DAA beginner. Symbol illustration: a, the distance from inferior AIIS to superior GT (abbreviated as D_GT-AIIS_/D_FH_); b, the distance from lateral ASIS to lateral GT; c, pelvic width max, the maximal distance between bilateral ASIS; d, pelvic width min, the minimal distance between bilateral teardrop. Iliac crest angle, the angle between lines connecting the AIIS and the ischial tuberosity

In addition to obesity, one result of Realyvasquez’s study that deserves our attention is the significant effect of GT/ASIS ratio (cut-off value of 1.17) on surgical time and intraoperative blood loss. This finding is actually in support of our previous study based on anatomical analysis and radiological assessment of typical bony landmarks around pelvis. In the study, we tried to use four indices as prominent variables of pelvic anatomy to establish indications for DAA. One index, termed as D_GT-AIIS_/D_FH_, is the ratio between the distance from inferior AIIS (anterior-inferior iliac spine) to superior GT and the diameter of the femoral head. The other three indices, designated GT/ASIS (the relative location of lateral GT to lateral anterior-superior iliac spine), PW_max_/PW_min_ (the ratio between the maximal pelvic width and the minimal pelvic width on the anteroposterior view of pelvis), and iliac crest angle, whose measurement is described in Fig. 1.

Besides, we also proposed a flexible approach in considering the indications and contraindications of DAA. In clinical practice, the DAA indications could range from simple, thin, less muscular, female femoral neck fracture, to complicated hip issues, such as severe dysplastic, stiff and ankylosed hips, even the conditions requiring one-stage total hip revision arthroplasty. A surgeon can handle a hip, with or without complicated issues, just as well when he is skillful in extensile surgical exposure/extension, soft-tissue release and tension retuning, bone defect reconstruction, as well as prothesis implanting and positioning in DAA procedure, *i.e*., the progression of DAA we reported in the aforementioned section in this perspective.

Moreover, we proposed a Xinqiao Predictive Algorithm for quantitative evaluation of the indications and contraindications based on the analysis of the high BMI and other stratified factors which may affect the decision-making, including waist-hip radio, comorbidities, pelvic anatomy, surgical complexity, and surgeon’s skills, and their cut-off value, data weight, and predictive power in determining indications level and feasibility in DAA are presented in Table 1.

**Table 1:** A Xinqiao-proposed predictive algorithm to determine indicative level and feasibility for DAA by a cumulative score method

**Indicator**	**Primary predictor**						**Second predictor**	
**Obesity**	30<BMI<35	+1	35<BMI<40	+1	BMI>40	+2	malnutrition	+0.5
	WHR<0.85 (male) or<0.9 (female)	+1	WHR>0.85 (male) Or > 0.9 (female)	+2	-	-	dermatitis	+0.5
**Pelvic Anatomy**	D_GT-AIIS_/D_FH_>1.5	0	0.5<D_GT-AIIS_/D_FH_<1.0	+1	D_GT-AIIS_/D_FH_<0.0	+2	diabetes mellitus	+0.5
	lateralized GT to ASIS	0	overlapped GT to ASIS	+1	medialized GT to ASIS	+2	hypothyroidism	+0.5
	PW_max_/PW_min_<2.0	0	PW_max_/PW_min_ >2.0	+1	PW_max_/PW_min_ >2.5	+2	vascular insufficiency	+0.5
	iliac crest angle<65°	0	iliac crest angle<75°	+1	iliac crest angle>75°	+2	immunosuppression	+0.5
**THA Complexity**	primary regular	0	Primary complex or partial revision	+1	total revision	+2	chronic cardiopulmonary comorbidity	+0.5
**Surgeon’s Proficiency**	DAA specialist (>1000 cases)	-1	DAA professional (>500 cases)	0	DAA beginner (<100 cases)	+1	previous surgery	+1
**Indicative Level**								
**Cumulative Score**	<3.5		3.5-6.0		>6.0			
**Risk & Complication**	Low		Medium		High			
**Indicative Level**	Strongly indicated		Fairly indicated		Conditionally indicated			
**Surgical Recommendation**	Standard DAA		Standard DAA + targeted soft tissue release + specific osteotomy		Extensile DAA + specific osteotomy + specific reconstruction			

### Advantages of DAA in surgical decision-making

The conspicuous advantages of DAA may include: (1) cosmetic benefits, such as small incision (including bikini incision), minimally-invasive and tissue-sparing surgery achieved via neuro-muscular interface [17]; (2) fast recovery, as promoted by less blood loss and pain, faster functional recovery in terms of muscle strength, range of motion, gait and early return to weight-bearing activities [4, 10]; (3) more favorable outcomes, including reduced dislocation rate and increased likelihood of components being placed into the safe zone [18]. If we further look into the issue, especially when a surgeon’s volume of DAA operation is more than 2000 cases, we will find that the advantages of DAA are beyond above-mentioned benefits to include the functional gain in hip-spine complex.

**Fig. 2:**
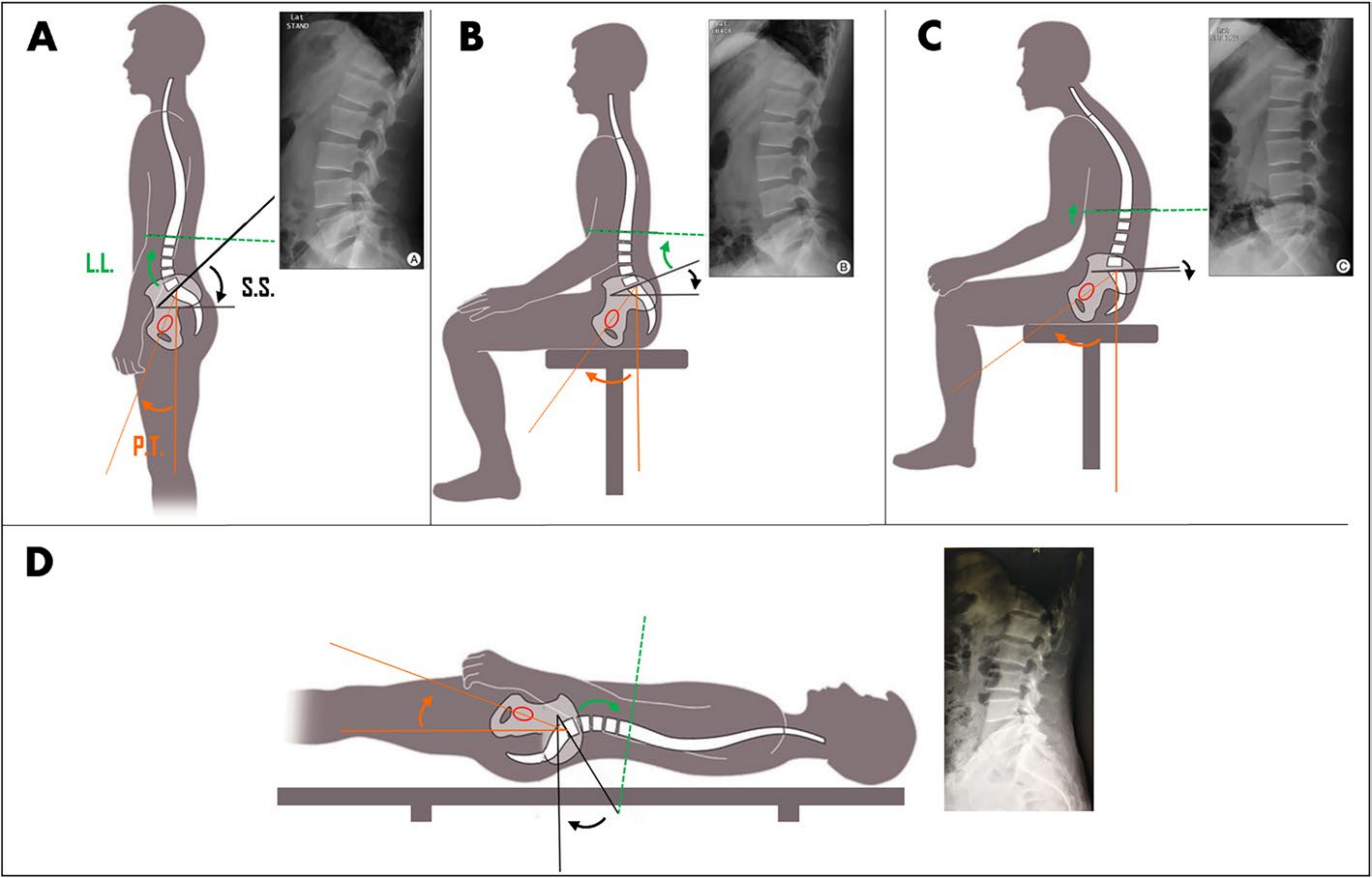
The hip-spine relation at different positions of standing, upright sitting, declined sitting, and supine position. **A** Typical parameters used for evaluating hip-spine coordination in standing, LL = 46°, SS = 43°, PT = 20°. **B** Typical parameters used for evaluating hip-spine coordination in upright sitting, LL = 24°, SS = 21°, PT = 35°. **C** Typical parameters used for evaluating hip-spine coordination in declined sitting, which imitates lateral decubitus position in traditional posterolateral approach THA, LL = 4°, SS = 2°, PT = 54°. **D** Typical parameters used for evaluating hip-spine coordination in supine position, which imitates supine DAA, LL = 40°, SS = 39°, PT = 20°

The concept of hip-spine complex received growing attention of surgeons in THA, especially in their decision-making in the management of complex hip disorders, and the causal analysis of failed arthroplasty due to instability or dislocation. The hip-spine relation is a dynamic mechanism through which coordinated movements of the spine, pelvis and hip work together to maintain balance on the coronal and sagittal planes of the body. Under physiological condition, lumbar lordosis (LL, 60° ± 10°) and pelvic anteversion (40° ± 10°) needed to increase acetabular coverage to remain stable in standing position, while spine straightening, pelvic retroversion (20° ± 9°), and hip flexion (132° ± 12°) occur when a person is sitting [19] (Fig. 2).

**Fig. 3:**
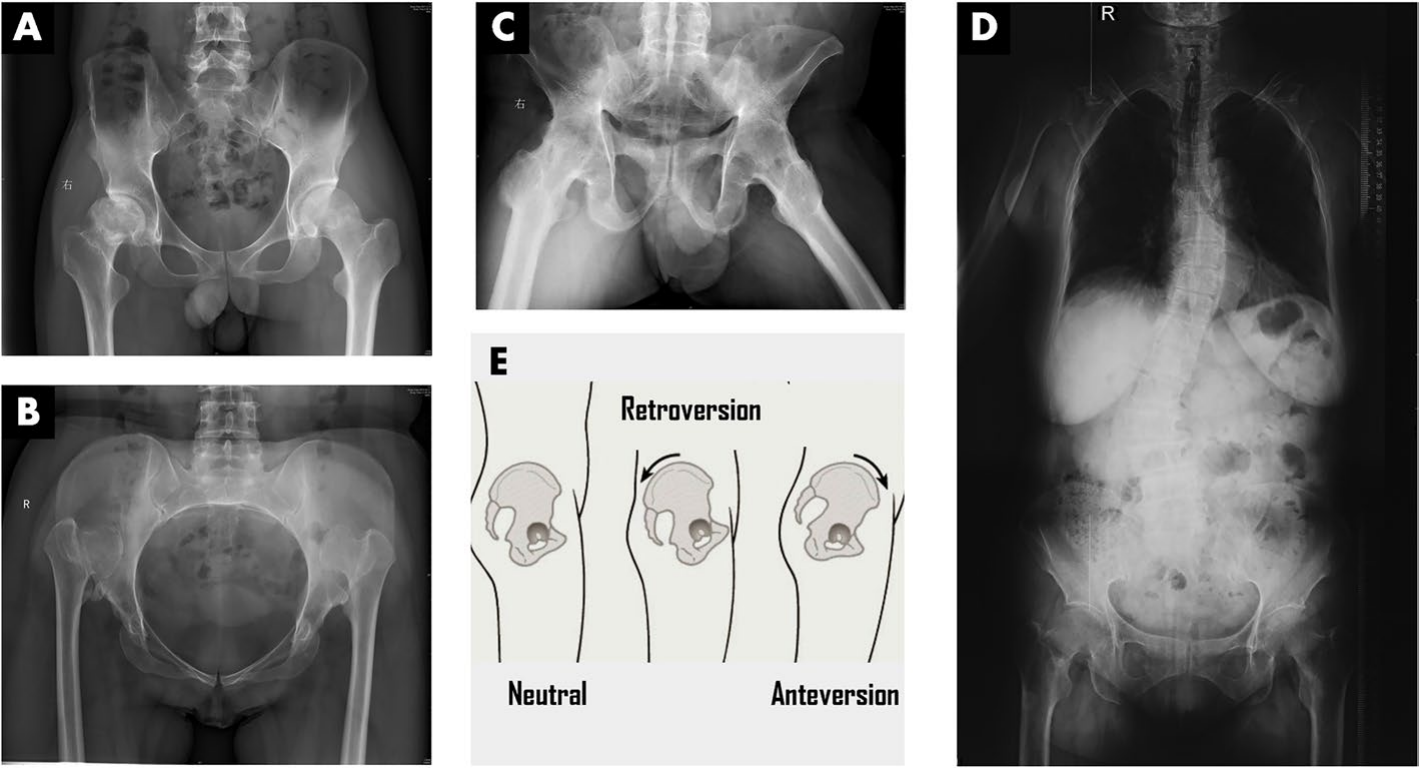
Anteroposterior view of a pelvic X-ray is a mirror reflecting different condition of hip-spine complex. **A** Bilateral femoral avascular necrosis with a normal pelvis orientation and physiological hip-spine relation, which constitutes low risk in THA. **B** Bilateral hip dislocation and osteoarthritis resulted from DDH with an extremely anteverted pelvis and hypermobile hip-spine relation, which constitutes high risks of instability, dislocation and mechanical loosening in THA. **C** Hip involvement of ankylosing spondylitis with an extremely retroverted pelvis and stiff hip-spine relation, which constitutes high risk of impingement, wear failure, and dislocation in THA. **D** Hip osteoarthritis secondary to degenerative lumbar disease with moderately retroverted pelvis and stiff hip-spine relation, which constitutes high risk of impingement, dislocation and abnormal gait in THA. **E** An illustrative manifestation of pelvis in lateral view showing neutral, retroverted and anteverted pelvis in above hip pathologies

**Fig. 4:**
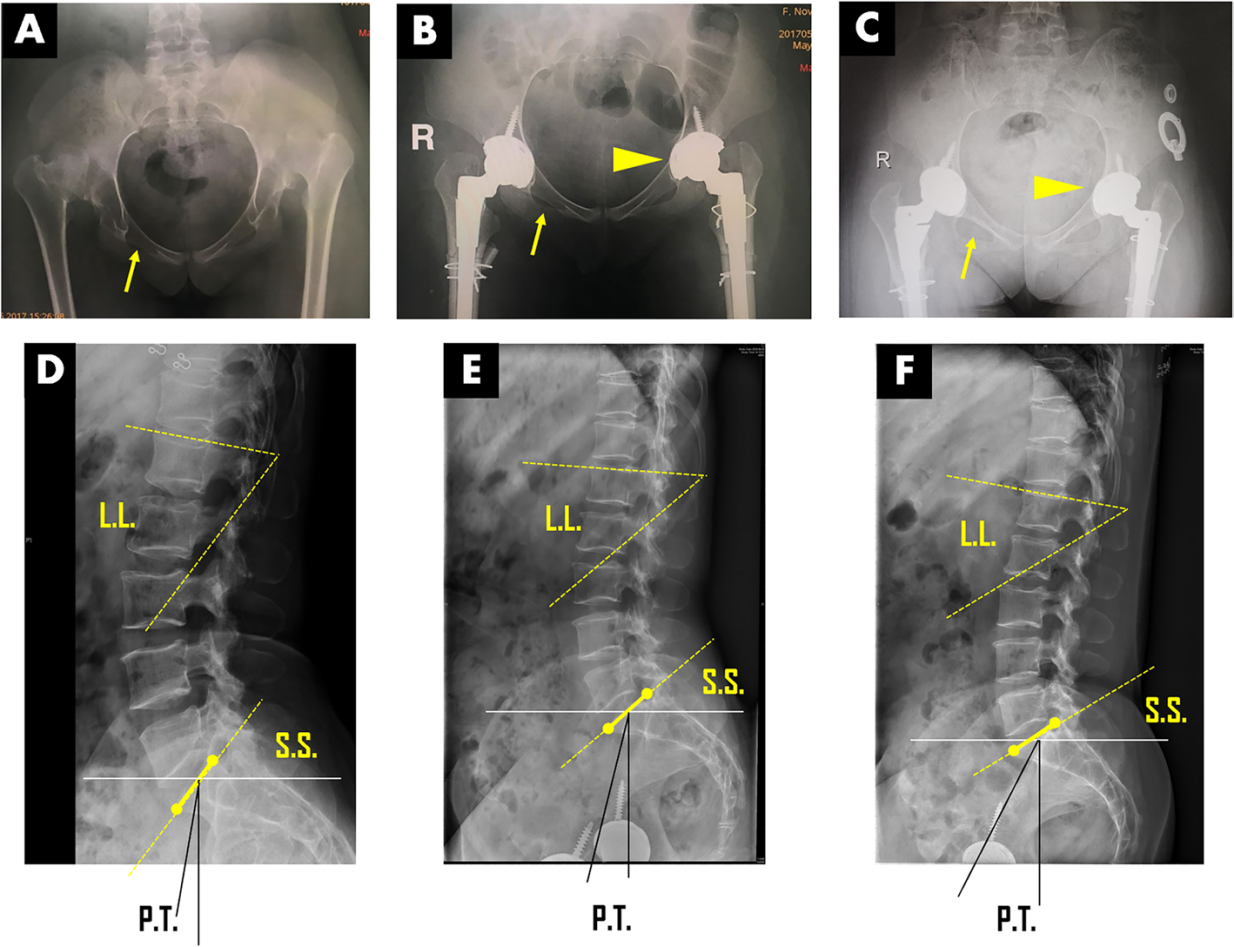
Specific temporal and spatial adaption observed from anteroposterior and lateral views of lumbar-pelvis X-ray in a bilateral high-dislocated DDH patient after DAA. **A**, **D** Preoperative anteroposterior pelvis X-ray demonstrated an extremely anteverted pelvis with opened inlet and closed obturator foramen (yellow arrow). The lateral X-ray of lumbar spine showed 64° of LL, 53° of SS, and 9° of PT. **B**, **E** Hip-spine adaption at the 6^th^ month after surgery, the anteroposterior pelvis X-ray showed slightly retroverted pelvis with opened obturator foramen (yellow triangle). And the inclination and anteversion angles of the acetabular component were 45° and 11° in right hip, and 38° and 9° in left hip. The lateral X-ray of lumbar spine showed 43° of LL, 40° of SS, and 14° of PT. **C**, **F** Hip-spine adaption at the 10^th^ month after surgery, the inclination and anteversion angles of the acetabular component transformed as 48° and 16° in the right, and 40° and 15° in the left (yellow triangle). The lateral X-ray of lumbar spine showed remodeled hip-spine complex with 42° of LL, 31° of SS, and 27° of PT. Please be noted the significant morphological change of obturator foramen (yellow arrow)

In actuality, the decompensation of hip-spine complex is far frequent than we discovered at present, especially in patients suffering from degenerative lumbar disease, multi-segmental lumbar fixation, developmental dysplasia of the hip (DDH), and spondyloarthropathy (such as rheumatoid arthritis and ankylosing spondylitis), *etc* [20, 21] (Fig. 3). It has to be emphasized that the hip-spinal complex undergoes specific temporal and spatial adaption in THA especially, in the above pathologies. For example, most patients with unilateral complex DDH anatomically have a coronal imbalance that manifests as a pelvic tilt toward the distal part of the limb of the dislocated hip, with little change in the sagittal balance of the pelvis. After anatomical reduction and correction of limb length inequality by THA, the pelvis gradually rotates on the coronal plane until restoring to the neutral position [22]. However, the bilateral DDH tends to have commitment severe sagittal decompensation, which presents as excessive pelvic anteversion, increased lumbar lordosis (LL) angle (normal: male 61.4° ± 10.2°, female 58.1° ± 10.8°), decreased pelvic incidence (PI) (normal: male 53.2° ± 10.3°, female 48.2° ± 7°), decreased sacral slope (SS) angle (normal: male 41.9° ± 8.7°, female 38.2° ± 7.8°), and decreased pelvic tilt (PT) angle (normal: male 11.9° ± 6.6°, female 10.3° ± 4.8°) [23]. Approximately 6 months after THA, adaptive change takes place in hip-spine complex upon sagittal correction of the pelvic anteversion to a neutral position, which is dubbed temporal and spatial change in the hip-spine relation (Fig. 4). The ignorance of temporal and spatial adaption will lead to loss of the functional safe zone of the components’ position, and eventually results in operative failure.

With this in mind, the differences between the original pelvic orientation and the functional position should be fully considered in THA to achieve the long-term survival of the prosthesis. One study examined the hip-spine relation under different conditions, including standing (hip extension with weight-bearing), supine position (hip extension without weight-bearing), upright sitting (60 degrees of hip flexion), and declined sitting (100 degrees of hip flexion), and found that the pelvic orientation in supine DAA was close to functional position in standing position, while lateral decubitus usually employed in traditional THA was identical to the retroverted pelvis when a person is sitting declined [24]. Therefore, supine DAA could be considered as an ideal position in deciding functional pelvic orientation and the ideal position of acetabular component, which plays a pivotal role in hip stability and bearing wear after THA. This is one key reason why we could reach high success rate in obtaining ideal component position in our supine DAA procedures without any intraoperative fluoroscopic verification.

## Conclusion

Chinese hip surgeons increasingly favor DAA with the volume of THA growing in recent years. In this article, we suggest that a surgeon using DAA should adjust his perspective in assessing indications/contraindications. The advantages of DAA go far beyond the minimal invasiveness and tissue sparing, and more potential advantages can be achieved by personalizing the pelvic functional position, reaching the genuine safe zone of acetabular component, adapting to the spatiotemporal change of the hip-spine coordination. However, well-designed, multi-center, prospective randomized controlled trials are warranted to provide robust evidence for this observational perspective.

## Data Availability

The datasets used and/or analyzed in the current study are available from the corresponding author on reasonable request.

## Abbreviations

DAA: Direct anterior approach
THA: Total hip arthroplasty
SSO: Subtrochanteric shortening osteotomy
BMI: Body mass index
ASIS: Anterior superior iliac spine
GT: Greater trochanter
WHR: Waist-hip ratio
AIIS: Anterior inferior iliac spine
DDH: Developmental dysplasia of the hip
LL: Lumbar lordosis
PI: Pelvic incidence
SS: Sacral slope
PT: Pelvic tilt
